# The interplay of suspended sediment concentration, particle size and fluid velocity on the rapid deposition of suspended iron oxide particles in PVC drinking water pipes

**DOI:** 10.1016/j.wroa.2022.100143

**Published:** 2022-04-15

**Authors:** Artur Sass Braga, Yves Filion

**Affiliations:** Department of Civil Engineering, Queen's University, 58 University Ave, Kingston, ON K7L 3N9, Canada

**Keywords:** Discolouration, Suspended Sediment Concentration, Particle Size, Iron Oxides, Particle Deposition

## Abstract

The inner walls of drinking water pipes are often expected to be clean and controlled surfaces to assure safe water access to the public. However, these surfaces are typically contaminated with particulate materials and biofilms that eventually degrade water quality. While water utilities place significant efforts in identifying and flushing material deposits from compromised pipes, the development of effective preventive strategies is still limited by the lack of knowledge about material accumulation processes. The aim of this paper is to examine the interplay between suspended sediment concentration, particle size and fluid velocity and the attachment of suspended iron oxide particles in PVC drinking water pipes. For that purpose, a series of short experiments were completed, whereby water amended with a known concentration of selected and stable iron oxide particles was introduced in a full-scale pipe loop laboratory over a short period of time and both turbidity and suspended sediment concentration (SSC) were measured at the inlet and outlet of the pipe loop. Results showed that a selected fraction of the injected particles with sizes above a specific threshold in the range of 4.6 to 6.8 µm had not reached the downstream section of the pipe loop, but instead remained attached to the pipe walls. In addition, exponential decay of the SSC was observed to occur along the pipes and to cause most of the sediments to accumulate in the upstream section of the pipe loop. The research improved our understanding of the physical processes of particulate material accumulation in DWDSs, and it will help water utilities forecast and monitor material accumulation and discolouration potential.

## Highlights

• Suspended sediment plume produced sediment deposits on PVC drinking water pipes.
• Iron oxide particles as small as 4.6 μm were rapidly deposited on pipe walls.
• Self-clean velocities were insufficient to prevent fine particle deposition.
• Deposition followed an exponential decay process along the length of the pipe.
• Adhesion forces enhanced by gravity likely selected particles that formed deposits.

## Graphical abstract

**Figure:**
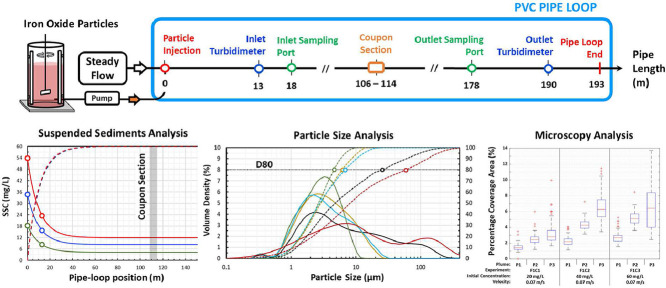

## 1 Introduction

The quality of finished drinking water is expected to deteriorate during its transport through pipes of drinking water distribution systems (DWDSs). The accumulation of material deposits inside the pipes of a distribution network is often suggested as a major factor that contributes to water quality deterioration (Vreeburg and Boxall, 2007). Material deposits can be resuspended and cause water to be discoloured (Husband and Boxall, 2011; Seth et al., 2004) and spur the growth of microorganisms and biofilms at the pipe wall (Lehtola et al., 2004; Vreeburg et al., 2008). Further, contamination with viable microorganisms (Liu et al., 2016), heavy metals (Liu et al., 2017), and antimicrobial genes (Rilstone et al., 2021) all have the potential to cause severe health implications. Despite this, water utilities often lack the tools and methods to adequately assess the build-up of material deposits in operational networks.

Traditionally, when water utilities suspect that a pipe section is harboring material deposits or contaminants that are degrading drinking water quality, they resort to flushing to mobilize materials and clean the culprit sections. In most cases, flushing effectively removes materials in the short term but it does not prevent new materials from accumulating on the pipe wall. Flushing operations are expensive also consume a substantial amount of water and require the isolation of pipe sections and service interruptions to customers. Therefore, strategies to slow the rate of material deposition on the wall of drinking water pipes in DWDSs can reduce the human and physical resources needed for flushing operations and contribute to maintaining safe drinking water.

Numerous studies have shown that particulate iron oxide is the dominant material that accumulates in DWDSs (Lehtola et al., 2004; Seth et al., 2004). Evidence from flushing old cast iron (CI) pipe mains suggests that most of the iron oxide particles are sourced from the internal corrosion of CI pipe since these sections typically generate the highest mobilized load in a single flushing operation (Boxall and Saul, 2005; Husband and Boxall, 2010). Research also shows that loose iron oxide particles are continually transported from their source components to the downstream regions of a DWDS (Husband et al., 2014). While the apparent load of iron oxide particles in non-metallic pipe sections is substantially smaller than that in their CI counterparts, iron oxides are still the dominant constituent of mobilized materials in non-metallic pipes (Seth et al., 2004). Nevertheless, Vreeburg et al. (2008) also found that pipe sections with higher inorganic material deposits fostered the growth of organic matter on the pipe wall which suggests that material deposits enhance biofilm growth.

In the last two decades, researchers have developed conceptual models to explain the mechanics of material deposit accumulation. Focusing on the relationship between turbidity and discolouration risk, Boxall et al. (2003) and Boxall and Saul (2005) suggested the use of a *turbidity potential* to quantify the materials that can be mobilized at the pipe wall. They proposed an empirical model named *Prediction of Discoloration Events in Distribution Systems* (PODDS) that simulates material accumulation as material layers with shear strength conditioned by the wall shear stress (WSS) of the flow at the pipe wall. In a flushing simulation, PODDS calculates the increase of the turbidity by comparing the WSS to the shear strength of each material layer to determine which layer will be mobilized. This concept has been validated by several field and laboratory studies (Boxall and Saul, 2005; Cook and Boxall, 2011; Husband and Boxall, 2010). Later, Furnass et al. (2014) reported the flaws of the PODDS regeneration module and suggested that material layers across all shear strengths may accumulate simultaneously and independently of the current WSS of the flow. Furnass et al. (2014) further suggested that this coefficient may change over time and its accurate determination still requires further investigation. Blokker and Schaap (2015) also proposed the estimation of material deposition on the basis of turbidity at the upstream inlet of a water main. They measured the turbidity-equivalent mass in controlled sections located upstream of an operational network and compared it to the turbidity-equivalent mass mobilized from flushing these sections.

Ryan et al. (2008) suggested that material deposition is comprised of two distinct mechanisms: the first is based on the gravitational settling of particles, and the second is based on the attachment of particles to the pipe wall. Here Ryan et al. (2008) developed a semi-empirical approach to simulate the settling of particles adapted from a sewer sediment transport model that makes use of information about particle size, particle specific density, and flow. For their system, a threshold velocity of 0.2 m s^-1^ was found to prevent deposition, while Vreeburg and Boxall (2007) proposed a pragmatic *self-cleaning velocity* threshold of 0.4 m s^-1^, and Blokker et al. (2011) suggested a value between 0.2 – 0.25 m s^-1^; both these velocities are based on experimental data from operational DWDSs. Later, van Summeren and Blokker (2017) built upon the gravitational model from Ryan et al. (2008) by adding the concept of sediment bed-load transport adapted from previous research on sediment transport in open channels. The authors identified a range of possible scenarios and conditions for bed-flow occurrence in DWDSs, but further experimental evidence is still required to confirm their model.

For the second mechanism, Ryan et al. (2008) developed an empirical model named the *Particle Sediment Model* (PSM) that can estimate both the deposition and mobilization of particles to pipe walls. In this model, they assumed that particles can attach to the pipe walls of their experimental system through van der Waals force. The inclusion of a surface adhesion force in their model established a link to the build-up of material layers with the specific shear strength included in the PODDS model. Ryan et al. (2008) used a small pipe loop facility to maintain a suspension of particles at a velocity of 0.6 m s^-1^ for two hours before adjusting the pump settings to switch to a lower fluid velocity. Conditioning velocities in the range of 0.07 – 0.3 m s^-1^ were tested. The results showed that the bulk water particle concentration decayed exponentially until a stable concentration was maintained in suspension, dependent on the pipe material and on fluid velocity. Ryan et al. (2008) suggested that a diffusion-like mechanism based on a suspended solids concentration (SSC) threshold that is function of the fluid velocity governed whether particles would be: (1) deposited on the pipe wall, (2) stable, or (3) resuspended from the pipe wall. In this context, Boxall and Saul (2005) showed that the average particle size of materials mobilized from flushing drinking water pipes was in the range of 10 µm, and they suggested that in most water mains in operational networks, turbulent conditions are enough to prevent gravitational settling of these particles. In addition, van Thienen et al. (2011) developed a theoretical model to explain the transport of suspended particles of different sizes under hydraulic conditions of DWDSs, but the model was not sufficient to explain the predominance of fine particles on samples from discoloured water.

Despite the many advances made in understanding material deposition mechanisms, several questions remain unanswered regarding how particles adhere to pipe walls and resist resuspension when subjected to wall shear stress. Current conceptual models do not explicitly include the particle size distribution and particle density. The lack of knowledge about the dynamics of these variables limits the accuracy of models based on gravitational settling due to the strong dependence of particle size on fall velocities; it also limits the accuracy of models that make use of a stable relationship between turbidity and suspended solids concentration (SSC). Furthermore, while cohesion is widely assumed to be related to biofilms, it is unclear which forces contribute to holding inorganic particles on the wall in the absence of biofilms. Previous experiments completed in a full-scale PVC pipe loop facility by Braga and Filion (2021) showed that fine iron oxide particle deposits were accumulated only on the pipe invert and that only a small fraction of particles were still held in suspension after a 30-day period where fluid velocity was held constant. Their results suggest that adhesion forces did not develop between particles and the pipe wall to hold them to the obvert or the springline positions of the pipe, and that particles as small as 1 µm were able to settle onto the pipe invert and resist the WSS of common operational velocities in DWDSs. These results were in stark contrast to previous research findings (Boxall and Saul, 2005) and demonstrate that further examination of the mechanisms behind the accumulation of sediment deposits is required.

This paper aims to examine the dynamics of acute particulate attachment on pipe walls (Sunny et al., 2020) following the rapid release and travel of a concentrated plume of iron oxide particles immediately following a simulated discolouration event in controlled experiments completed in a full-scale PVC pipe loop laboratory. The specific objectives of the experiments were to: (1) estimate the average rate of iron oxide particle deposition on PVC pipes immediately after the rapid release, and during the passage of, a highly-concentrated plume of iron oxide particles that typifies discolouration events in real systems; (2) determine whether previously deposited particles on the pipe wall from the passage of previous iron oxide particle plumes affect the average rate of iron oxide deposition and attachment to the pipe wall, and (3) examine the specific mechanisms involved in the particle settling phenomenon for drinking water pipes, and the effect of particle size distribution of sediments in suspension on the formation of material deposits on the pipe wall.

## 2 Methods

**Fig. 1:**
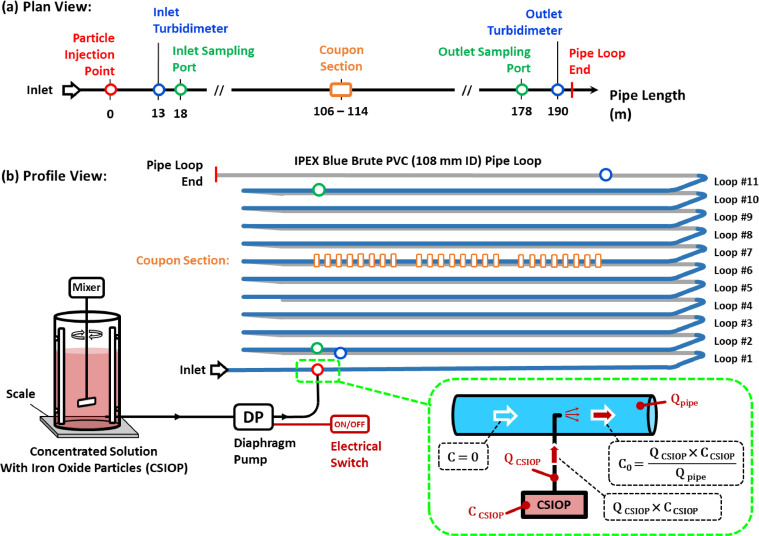
Schematic of the full-scale pipe loop of the Drinking Water Distribution Laboratory at Queen's University. The diagram features the injection of iron oxide particles in the system using a concentrated solution and a diaphragm pump, and the position of the sampling ports, turbidimeters and a coupon sample section in the system.

The experimental work was realized in the *Drinking Water Distribution Laboratory* (DWDL) at Queen's University, using a full-scale pipe loop rig that simulates the operation of drinking water mains. The pipe laboratory system is comprised of a water tank with a volume of 3.6 m³, two variable speed centrifugal pumps and 11 loops of IPEX Blue Brute PVC pipe Class 235 (DR18) with an internal diameter of 108 mm, and a total length of 193 m (Fig. 1). During the experiments, the pipe loop system was operated in a non-recirculatory manner, where drinking water from the City of Kington was continuously added to the tank, pumped through the pipe system, and discarded at the end. The water from the City of Kingston has a pH of 8.1, a hardness of 123 mg L^-1^-CaCO_3_ and an alkalinity of 92 mg L^-1^-CaCO_3_, and has approximately 1.7 mg L^-1^ of dissolved carbon and 1.0 mg L^-1^ of total nitrogen (Utilities Kingston, 2017). During the experiments the water temperature ranged between 12°C and 14°C.

In the experiments, drinking water was amended with iron oxide particles of known particle size distribution at the inlet to the pipe loop to induce the formation of material deposits on the pipe walls. A total of five experiments were performed with a variable concentration of suspended particles (F1C1, F2C2 and F1C3 in Table 1) and a variable conditioning flow rate (F1C1, F2C1 and F3C1 in Table 1). During all the experiments, the pipe flow rate and turbidity were continuously monitored at a sampling frequency of 1 Hz using two Sierra InnovaSonic® 205i Ultrasonic Flow Meters (±0.5%) and two online Hach TU5300sc turbidimeters (±0.01 NTU) (Fig. 1). The turbidimeters were configured to monitor turbidity at the inlet (7% of the pipe length) and outlet (98% of the pipe length) (Fig. 1). Turbidity was measured from water continuously sampled from the centre of the pipe at a constant sampling flow rate. Only steady flow conditions were tested, but due to the short duration of the experiments, these flows can be interpreted as the highest daily peak flows of operational systems commonly used to determine the conditioning WSS in systems with variable demands (Husband and Boxall, 2011; Husband et al., 2008).

**Table 1:** Target design conditions for the experiments.

*Experiment*	*Concentration (mg L*^-1^*)*	*Flow (L s*^-1^*)*	*Velocity (*m s^-1^*)*	*Reynolds Number*	*WSS*^a^ *(Pa)*	*VSL*^b^ *(µm)*	*Pipe loop RT*^c^ *(min)*
*F1C1*	*20.0*	*0.60*	*0.07*	*7 000*	*0.02*	*1172*	*50.9*
*F1C2*	*40.0*	*0.60*	*0.07*	*7 000*	*0.02*	*1172*	*50.9*
*F1C3*	*60.0*	*0.60*	*0.07*	*7 000*	*0.02*	*1172*	*50.9*
*F2C1*	*20.0*	*1.80*	*0.20*	*21 000*	*0.11*	*450*	*17.0*
*F3C1*	*20.0*	*2.75*	*0.30*	*32 000*	*0.24*	*310*	*11.1*

a . Wall shear stress based on the Darcy-Weisbach equation.

b . Viscous sub-layer thickness.

c . Pipe loop residence time (RT) calculated by dividing the pipe loop volume of 1.7 m^3^ by the flow rate.

The conditioning stage of each experiment was divided in three independent sub-stages (P1, P2 and P3), where plumes of suspended particles were produced at the inlet of the pipe loop and completely transported through the pipe loop outlet before the introduction of the next plume in the next sub-stage. Each plume was created by injecting a known volume of a *concentrated solution of iron oxide particles* (CSIOP) at the inlet of the pipe loop with a known SSC ($C_{CSIOP}$, mg L^−1^) over an injection period equal to the time required for the water to travel 60 m downstream of the injection point given the prevailing flow rate ($Q_{pipe}$ , L s^-1^) in the pipe loop (Fig. 1). The plume length of 60 m was mainly defined by a limitation of the maximum injection volume under stable mixing conditions in the CSIOP. A new CSIOP was prepared for each plume, by addinga known mass of *iron oxide particles* (IOP) ($M_{IOP}$, mg) into a known drinking water volume ($Vol_{CSIOP}$ , L) in a mixing baffled tank operated at a fixed mixing intensity (Fig. 1). After the IOP addition in the tank, a period of 5 minutes was used to allow the particle suspension to stabilize in the mixing tank. A diaphragm pump was used to pump water from the bottom of the CSIOP tank (approximately 10 cm away from the tank walls) from a position of intense mixing between two of the four vertical baffles, and inject it at a constant flow at the inlet of the pipe loop, at the centreline cross-sectional position of the pipe (Fig. 1).

The final SSC of the CSIOP ($C_{CSIOP}$ , mg L^−1^) was measured immediately before (after the stabilization period) and immediately after the injection period by collecting grab samples from sampling tubing located in an equivalent position to the inlet of the diaphragm pump, and with a similar flow rate as the diaphragm pump. The injection flow rate ($Q_{CSIOP}$ , L s^−1^) was measured during the injection period by monitoring the decrease of mass in the starting solution in the baffled mixing tank using a conventional scale (Fig. 1). The SSC of the sediment plume generated from the mass injection at the start of the pipe loop ($C_{0}$ , mg L^-1^) was calculated using direct measurements from experimental variables through a mass balance relationship (Fig. 1 and Equation 1).

(1) $$C_{0}=\;\frac{Q_{CSIOP}\cdot C_{CSIOP}}{Q_{pipe}};C_{CSIOP}\approx \frac{M_{IOP}}{Vol_{CSIOP}}$$

This method for estimating the initial SSC was preferred to a direct grab sample collection from the pipe loop immediately after the injection because a mixing length is required to achieve a suspension stability in the pipe. Therefore, initial SSC in the pipes was estimated on the basis of a stable particle suspension in the CSIOP established after the stabilization period of 5 minutes during the short injection periods, given the constant mixing intensity and small variation in the water level in the tank. It is also reasonable to assume that the grab sample had an equivalent SSC to the CSIOP fraction injected in the pipe since the sample was collected from an equivalent location in the mixing tank, which is expected to have a flow symmetry around the vertical rotation axis, which produces similar particle suspension in both water intake regions (Cekinski et al., 2010). The average of the SSC difference between CSIOP samples collected before and after the injection was 3% which suggests that no changes occurred during the injection period. Lastly, the average uncertainty of the final SSC estimated at the pipe inlet was of 10%, while the average uncertainty of the SSC from samples collected directly at the pipes (presented in sequence) was 5%.

During the experiments, the three sequential injections with fixed conditions were designed to identify if the particle accumulation rate changed over successive plumes. It was hypothesized that the particle accumulation rate of the 2^nd^ and 3^rd^ plumes might be affected by the presence of particles deposited on the pipe wall after the first plume. The basis for this hypothesis is that the particles from the first plume might produce new features in the microscopic surface of the PVC pipe which might enhance the attachment of the new particles . At the end of each injection period, the steady flow conditions were maintained in the pipe loop for an additional period required to refresh the equivalent of 1.5 pipe loop volumes to guarantee that all suspended particles from the sediment plume had exited the pipe loop. Between each of the different experimental conditions tested, the pipe system was cleaned at the highest flowrate (16 L s^−1^) and clean coupon pipe wall samples (presented in sequence) were added after the cleaning flush to re-establish identical initial condition for all the experiments*.*

A chemical grade of red iron oxide powder from Alpha Chemicals, with a composition of 82% of Iron (III) oxide – Fe_2_O_3_, was chosen as the source of particles for the experiments due their representativeness of iron oxide particles found in DWDSs and their stable particulate form and insolubility in water. The powder was sent to a commercial laboratory to split it into sub-samples of 50 g with identical particle size distributions (PSD) using a mini rotary splitter from Retsch company model PT100. The PSD of two sub-samples picked at random was analyzed with a Malvern Mastersizer 3000 particle size analyzer (laser diffraction). In the preparation of the concentrated starting solution, 1 to 3 sub-samples were added to the CSIOP tank with a steady mixing rotation. The insolubility of the particles and negligible amounts of dissolved iron in the local drinking water guaranteed that particles remained stable during the experiments.

Following each injection, grab samples were collected at the inlet and outlet sampling port locations (Fig. 1) to determine SSC at those locations and to assess changes in the particles in suspension after the passage of each plume. The collection of grab samples was synchronized with the fluid velocity in the pipe loop to collect 4 L of water from the centre of the passing sediment plume. The SSC of the grab samples was determined using a dry-weight method, by filtering the sample volume with pre-weighed 0.45 µm glass microfiber filters, drying the filters at 105°C for 1 hour, and weighing them again after drying with a precision scale. Additional grab samples were also collected from the CSIOP and from the outlet sampling port during the passage of the third plume in experiments F1C1, F2C1 and F3C1 to assess changes of the PSD of the particles in suspension. The PSD of the grab samples was determined with the Malvern Mastersizer 3000 particle size analyzer.

A pipe wall coupon sampling system described in Braga and Filion (2021) was also used in the experiments to characterize the accumulation of material deposits at the pipe wall. The system allows for the collection of representative pipe wall coupon samples from a *coupon section* located at 55% of the pipe loop length (Fig. 1). The coupon samples consist of circular PVC disks that are inserted directly into custom pre-drilled holes in the PVC pipe loop. A precise surface alignment of the internal surface of the coupon with the surrounding pipe wall surface (in the range of ± 0.1 mm) guarantees that the coupon sample experiences the same hydrodynamic conditions as the remainder of the PVC pipe wall, without disturbing the local velocity profile in the viscous sub-layer region (VSL). Coupon samples were acquired between the passage of each plume and analyzed using brightfield microscopy to visualize the deposits of iron oxide particles on the internal surface of the PVC pipe. One hundred (100) field of views (FOV) of each coupon sample were captured using the Nikon Eclipse Ni-E automated upright microscope in brightfield mode at a final magnification of 400X. A previously developed MATLAB image-processing script was used to calculate the percent coverage sediment (%) and particle size analysis (PSA) of material deposits detected on the pipe coupons (Braga and Filion 2021).

## 3 Results

### 3.1 Material deposition estimation

**Fig. 2:**
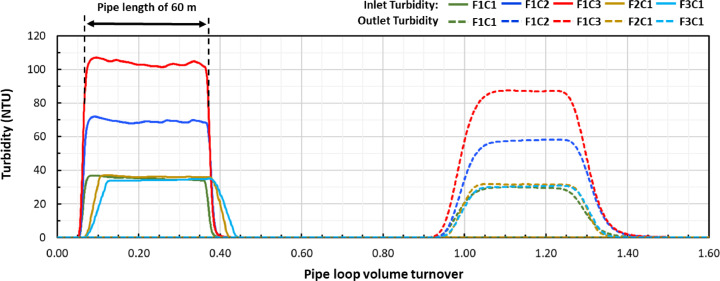
Turbidity of the moving sediment plume plotted against pipe loop volume turnover measured at the inlet and outlet turbidimeter locations. All sediment plumes were generated by injecting a known mass of iron oxide particles in a water volume equivalent that occupies a 60 m length of pipe.

Fig. 2 presents the turbidity measured at the inlet and outlet of the pipe loop against pipe loop volume turnover across the five experiments. The outlet turbidity profiles deviate from an “instantaneous” square shape likely due to a non-uniform velocity profile that caused some mechanical dispersion at the plume front and rear. This is also noticeable for the inlet turbidity data in experiments F2C1 and F3C1 realized at higher velocities (Fig. 2). Turbidity values as high as 100 NTU were measured in experiment F1C3. Similar turbidity values have been reported in operational DWDSs during intense discolouration events, but usually at higher flow rates (Husband and Boxall, 2010).

**Fig. 3:**
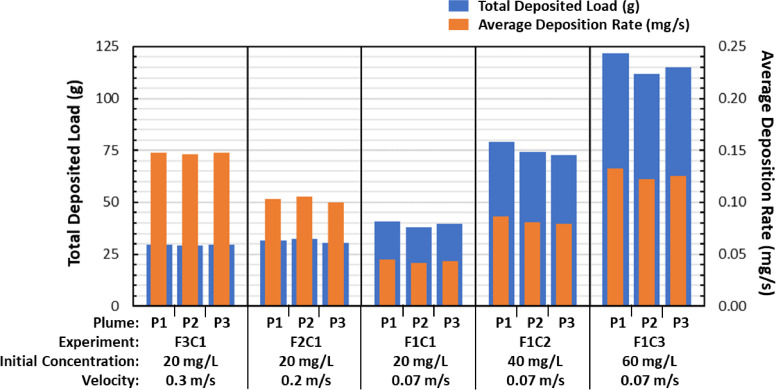
Total deposited load and average deposition rate for each plume of each experiment performed at three different velocities and similar concentration (F3C1, F2C1 and F1C1), and at three different initial concentrations and similar velocity (F1C1, F1C2 and F1C3).

Fig. 3 and Table 2 show the detailed results of the particle load estimations for individual plumes of each experiment. Table 2 indicates the estimated values of the SSC and the *total material load* (TML) at the three control sections of the pipe loop: (1) *injection point* (L = 0 m)*,* (2) *inlet turbidity meter* (L = 13.5 m), and (3) *outlet turbidity meter* (L = 189 m). A consistent decrease of SSC from the injection point to the *outlet* location demonstrates that a sizable fraction of the particles in suspension was deposited on the pipe walls of the system. The *total material load* at the *injection* location (TML_0_, g) was calculated by multiplying the *CSIOP* ($C_{CSIOP}$ , mg L^−1^) by the *injection flow rate* ($Q_{CSIOP}$ , L min^−1^) and the *injection period*. For the case of the *inlet* and *outlet* locations, the corresponding TML_IN_ (g) and TML_OUT_ (g) were estimated based on both the turbidity and SSC grab sample data, through a series of steps: 1) the turbidity measurements (NTU) were converted to an equivalent SSC (mg L^-1^) using a specific *linear regression coefficient* (mg L^-1^ NTU^-1^) presented later in this paper; 2) an instantaneous suspended sediment flux (mg s^-1^) was calculated by multiplying the SSC (mg L^-1^) by the flow rate (L s^-1^); and 3) the TML (g) was calculated by integrating the material flux over the total time required for the plume to travel through the pipe loop. The *total deposited load* (TDL, g) was calculated by taking the difference between the total material load at the injection point (TML_0_, g) and the total material load at the outlet (TML_OUT_, g).

**Table 2:** Particle load estimations for individual plumes (P1, P2 and P3) of each experiment, at three control sections of the pipe loop: injection point (0), inlet (IN), outlet (OUT).

E-P a	Q_CSIOP_^b^ (L min^-1^)	C_0_^c^ (mg L^-1^)	C_IN_ (mg L^-1^)	C_OUT_ (mg L^-1^)	TML_0_^d^ (g)	TML_IN_ (g)	TML_OUT_ (g)	TDL e (g)	TDL/ TML_0_ (%)
F1C1-P1	1.51 ± 0.3	18.9 ± 4.1	8.4 ± 0.3	4.3 ± 0.3	10.4 ± 2.1	4.7 ± 0.6	2.5 ± 0.5	7.8 ± 2.5	75.5%
F1C1-P2	1.36 ± 0.1	17.8 ± 2.2	7.9 ± 0.3	4.0 ± 0.3	9.7 ± 1.1	4.4 ± 0.5	2.4 ± 0.4	7.3 ± 1.5	75.3%
F1C1-P3	1.42 ± 0.1	18.5 ± 1.9	8.8 ± 0.3	4 3 ± 0.3	10.1 ± 0.9	4.7 ± 0.6	2.5 ± 0.5	7.6 ± 1.3	75.2%
F1C2-P1	1.37 ± 0.1	35.6 ± 3.5	15.3 ± 0.4	8.1 ± 0.5	20.1 ± 1.6	9.0 ± 1.1	4.9 ± 0.9	15.2 ± 2.5	75.8%
F1C2-P2	1.43 ± 0.1	34.4 ± 2.6	16.6 ± 0.5	8.7 ± 0.5	19.3 ± 1.1	9.4 ± 1.2	5.0 ± 0.9	14.3 ± 2.1	73.9%
F1C2-P3	1.42 ± 0.1	34.1 ± 2.9	15.8 ± 0.5	7.9 ± 0.5	19.0 ± 1.3	9.5 ± 1.1	5.0 ± 0.9	14.0 ± 2.2	73.6%
F1C3-P1	1.40 ± 0.2	55.6 ± 9.7	25.2 ± 0.6	12.3 ± 1.0	30.6 ± 4.9	13.7 ± 1.6	7.3 ± 1.3	23.3 ± 6.2	76.3%
F1C3-P2	1.37 ± 0.3	52.1 ± 11	23.2 ± 1.0	12.6 ± 1.0	29.1 ± 5.7	14.0 ± 1.7	7.6 ± 1.4	21.5 ± 7.1	73.8%
F1C3-P3	1.39 ± 0.3	53.8 ± 12	22.3 ± 1.0	10.8 ± 1.0	29.4 ± 6.3	13.7 ± 1.7	7.3 ± 1.3	22.1 ± 7.6	75.1%
F2C1-P1	1.24 ± 0.1	15.8 ± 0.7	9.9 ± 0.3	6.0 ± 0.3	8.7 ± 0.3	4.9 ± 0.6	2.7 ± 0.5	6.0 ± 0.8	69.2%
F2C1-P2	1.27 ± 0.1	16.0 ± 0.9	9.8 ± 0.4	5.2 ± 0.4	8.8 ± 0.4	4.8 ± 0.6	2.6 ± 0.5	6.2 ± 0.9	70.5%
F2C1-P3	1.28 ± 0.1	15.4 ± 1.0	9.5 ± 0.4	5.3 ± 0.4	8.5 ± 0.5	4.7 ± 0.6	2.6 ± 0.5	5.9 ± 0.9	69.4%
F3C1-P1	1.16 ± 0.1	14.7 ± 0.9	9.2 ± 0.3	5.8 ± 0.3	8.1 ± 0.5	4.4 ± 0.5	2.5 ± 0.4	5.7 ± 0.9	69.6%
F3C1-P2	1.19 ± 0.1	14.9 ± 1.0	9.8 ± 0.3	6.1 ± 0.3	8.2 ± 0.5	4.6 ± 0.5	2.5 ± 0.5	5.6 ± 1.0	68.9%
F3C1-P3	1.23 ± 0.1	15.1 ± 0.9	10.0 ± 0.4	5.9 ± 0.4	8.3 ± 0.5	4.7 ± 0.5	2.6 ± 0.5	5.7 ± 0.9	68.6%

a : Experiment – Plume.

b : CSIOP injection flow at the start section of the pipe loop.

c : Suspended sediment concentration.

d : Total material load.

e : Total deposited load.

Fig. 3 shows that large differences in TDL occurred between experiments performed at different concentrations (F1C1, F1C2 and F1C3), while only small differences were found between experiments performed at different velocities (F3C1, F2C1 and F1C1). Systematic variations of the particle deposition rate and attachment were not observed along the three plumes of each experiment (Fig. 3), suggesting that the presence of particle deposits from previous plumes had a negligible impact on particle deposition and attachment. The secondary vertical axis (right hand side of Fig. 3) shows that both an increase of concentration and fluid velocity produced an increase in the average deposition rate. Such increase was mainly caused by the shorter conditioning period of the experiments at higher velocities. Lastly, Table 2 also shows the fraction of the particles that were introduced in the pipe loop and were subsequently deposited onto the pipe walls (TDL/TML_0_) ranged from a minimum of a 69.2% in the F2C1-P1 experiment to a maximum of 76.3% in the F1C3-P1 experiment.

**Fig. 4:**
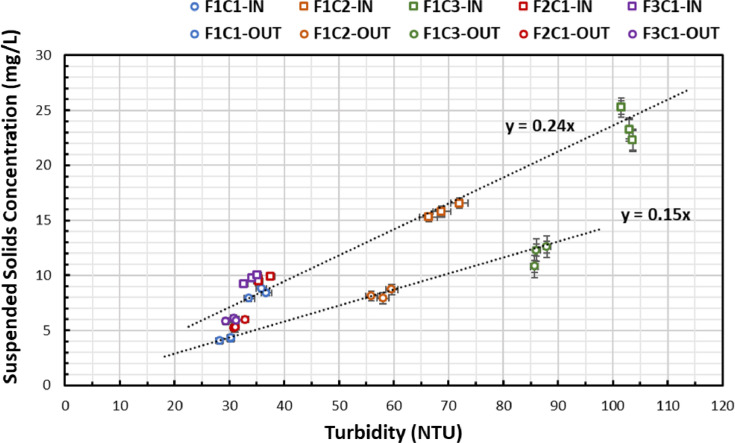
Linear regression relationships between turbidity and suspended solids concentration (SSC) for grab samples collected at the inlet and outlet locations.

A number of regression relationships were developed to relate measured turbidity to measured SSC in the pipe loop. The *linear regression coefficients* used to transform measured turbidity into SSC were obtained by performing a linear regression of the SSC measured from collected grab samples and their corresponding turbidity values (Fig. 4). The *linear regression coefficient values* of 0.24 mg L^-1^ NTU^-1^ and 0.15 mg L^-1^ NTU^-1^ determined through regression analysis are smaller than those in the research literature that reported turbidity values exceeding 1 mg L^-1^ NTU^-1^ in flushing operations (Pourcel et al., 2020).

### 3.2 Particle size analysis

**Fig. 5:**
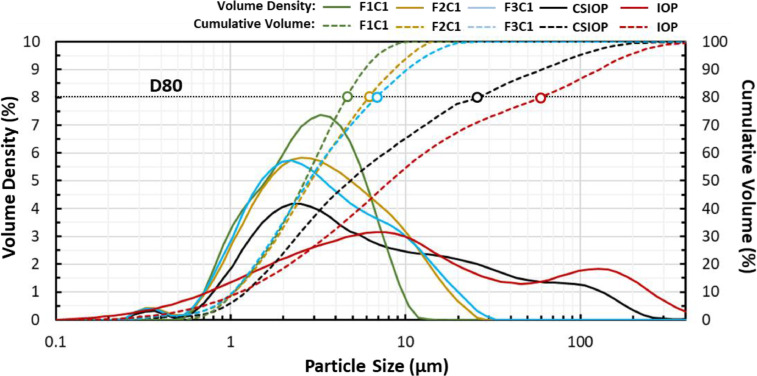
Particle size distribution of the initial iron oxide particle powder (IOP), the concentrated iron oxide particle starting solution (CSIOP) and samples collected at the outlet location during the third wave of the F1C2, F2C1 and F3C1 experiments. The primary vertical axis (left-hand side) denotes the volume density highlighting the predominant particle size, and the secondary vertical axis (right-hand side) denotes the cumulative volume which corresponds to the particle volume which is below a specific particle size.

The paper also examined the impact of fluid velocity on the evolution of iron oxide particle size distribution in the experiments. Fig. 5 plots the particle size distribution measured from samples collected at different experimental stages, including: 1) the initial iron oxide particles (IOP) used to prepare the CSIOP; 2) the suspended particles from the CSIOP mixing tank that were injected into the pipe loop; and 3) the particles in suspension at the outlet of the pipe loop during the passage of the third sediment plume in experiments F1C1-P3, F2C1-P3, and F3C1-P3 where the fluid velocity was increased from 0.07 m s^-1^ to 0.3 m s^-1^. In the plots of Fig. 5, the v*olume density (%)* on the primary vertical axis (left-hand side) denotes the percentage of the total particle volume for each particle size, and the *cumulative volume (%)* on the secondary vertical axis (right-hand side) corresponds to the value of particle size below which a certain percentage of the sample lies. The volume density curves show the predominant particle sizes of each distribution (peak of curves), and the particle size range of a sample. The volume density curves in Fig. 5 have peaks that are below 10 µm which indicates a predominance of small particle sizes in all the experimental stages. However, a large upper range of particle sizes was found for the IOP and CSIOP samples with particles as large as 100 µm, while the particle size range at the outlet of the pipe loop was limited to 10 µm for the F1C1 experiment, and 20 µm for the F2C1 and F3C1 experiments.

The cumulative volume curves in Fig. 5 show that the initial IOP powder had a D80 (80% of the particle volume is below a specific particle size) of 60 μm and that this was reduced to 26 μm at the CSIOP mixing tank. These results suggest that even with an intense level of mixing in the CSIOP baffled tank, a large fraction of particles had settled out of solution in the mixing tank before they were introduced in the pipe loop. During the passage of the sediment plumes in the pipes, the particles in suspension at the injection point had a D80 of 26 μm, while the particles in suspension at the outlet had a D80 of 4.6 μm in the F1C1 experiment (velocity of 0.07 m s^-1^), 6.2 μm in the F2C1 experiment (velocity of 0.2 m s^-1^), and 6.8 μm in the F3C1 experiment (velocity of 0.3 m s^-1^). The differences in the D80 between the injection point and the outlet of the pipe loop suggest that particles in the range of 4.6 μm – 26 μm were deposited in the F1C1 experiment, particles in the range of 6.2 μm – 26 μm in the F2C1 experiment, and particles in the range of 6.8 μm – 26 μm in the F3C1 experiment. Therefore, the lower limit of the sizes of the particles that are deposited establishes a *particle size threshold*. Particles with sizes larger than this threshold will be deposited on the pipes (d*eposited particles* – DP), while particles with sizes smaller than the threshold will be maintained in suspension (*non-deposited particles* – NDP). It is clear that the increase in fluid velocity raised the *particle size threshold* and kept larger particles in suspension as observed with the decrease of the DP fraction. These findings are in agreement with the decrease in the deposited materials observed across experiments F2C1 and F3C1 where the fluid velocity was increased from 0.2 m s^-1^ to 0.3 m s^-1^ (Table 2).

In addition, using the CSIOP volume density curve of Fig. 5, it is possible to estimate the fraction of the injected particles that corresponds to a determined particle size range (partial cumulative volume). Choosing a particle size range from 4.6 µm to 6.8 µm, defined by the *particle size threshold* of the two experiments with the larger velocity difference (F1C1-P3 and F3C1-P3), results in a CSIOP partial cumulative volume of 6.5%, which represents the fraction of the injected particles (CSIOP) that were deposited in the experiment with the lower velocity (F1C1-P3) but were kept in suspension in the experiment with the higher velocity (F3C1-P3). In other words, that is the fraction of the initial injected particles that changed between DP to NDP fractions as consequence of the increase in flow velocity from 0.07 m s^-1^ to 0.3 m s^-1^. By comparison, the proportional reduction of the TDL (Table 2) between the experiments F1C1–P3 and F3C1–P3 was estimated to be 6.6%. The close agreement between the two metrics (estimated from different experimental measurements) is strong evidence that the reduction of TDL between the experiments performed at fluid velocities 0.07 m s^-1^ and 0.3 m s^-1^ was caused by the non-deposition of particles in the range of 4.6 µm to 6.8 µm.

### 3.3 Suspended sediment decay model

**Fig. 6:**
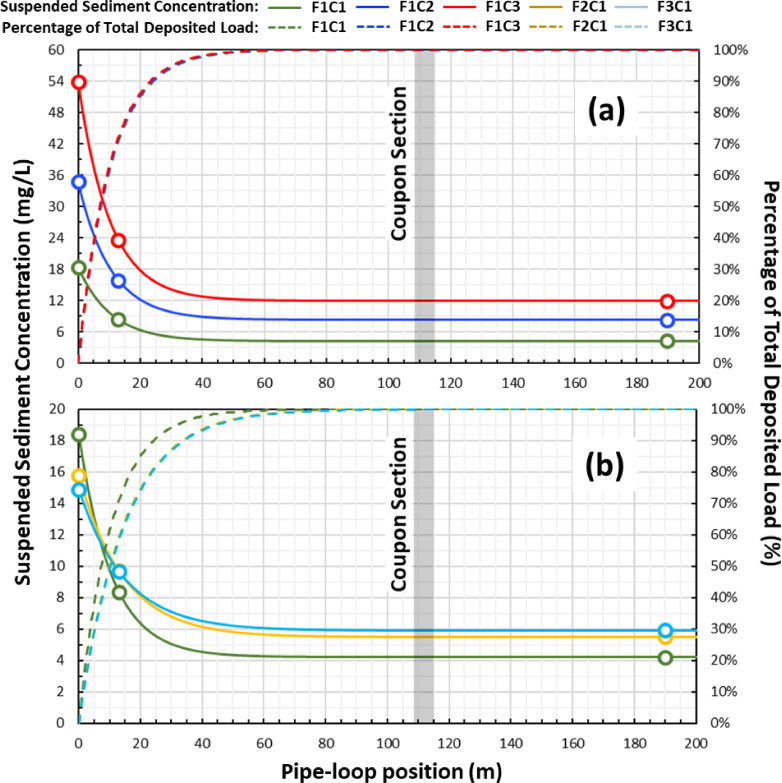
Average decay of suspended sediment concentration of the sediment plumes along their passage through the pipe loop and percentage of total deposited load for the (a) three experiments F1C1, F1C2, and F1C3 performed with a fluid velocity of 0.07 m s^-1^ and injection concentrations of 20 mg L^-1^, 40 mg L^-1^, and 60 mg L^-1^, and (b) three experiments F1C1, F2C1, and F3C1 performed with an injection concentration of 20 mg L^-1^ and fluid velocities of 0.07 m s^-1^, 0.2 m s^-1^ and 0.3 m s^-1^.

**Fig. 7:**
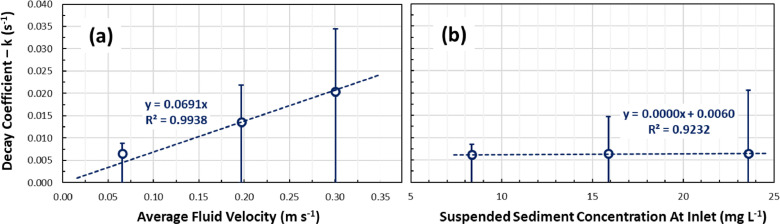
Concentration decay coefficient for the (a) three experiments F1C1, F2C1, and F3C1 performed with an injection concentration of 20 mg L^-1^ and fluid velocities of 0.07 m s^−1^, 0.2 m s^−1^ and 0.3 m s^−1^, and (b) three experiments F1C1, F1C2, and F1C3 performed with a fluid velocity of 0.07 m s^−1^ and injection concentrations of 20 mg L^-1^, 40 mg L^-1^, and 60 mg L^-1^.

The evolution of particle deposition along the length of the pipe loop was also examined. Fig. 6 plots the SSC measured at the injection point, the inlet, and outlet of the pipe loop with their corresponding pipe length positions. The SSC data points were found to conform to a first-order exponential decay process which is indicated with solid lines in Fig. 6. The first-order exponential decay model in Equation 2 was used to characterize the decrease in SSC along the pipe length as particles are continuously deposited onto the pipe wall.

(2) $$C\left(x\right)=\;C_{DP}\;e^{-\left(\frac{k\;x}{v}\right)}+C_{NDP}\;∴\;C_{0}\;=\;C_{DP}+C_{NDP}$$

where C(x) (mg L^-1^) is the SSC measured at pipe length location x (m), $C_{DP}$ (mg L^-1^) and $C_{NDP}$ (mg L^-1^) are the DP and NDP components of the SSC measured at the injection point – $C_{0}$ (mg L^-1^), k (s ^-1^) is the decay coefficient and v (m s^-1^) is the fluid velocity in the pipe loop. The solid lines in Fig. 6 demonstrate that a stable SSC is established after the mid-point of the pipe loop length, which corresponds to the NDP component of the initial SSC (C_NDP_, mg L^-1^). The NDP fraction does not contribute to the formation of material deposits and therefore sets a baseline for the SSC in each experiment. The decay coefficient (k), which governs how fast the DP fraction of the initial SSC is deposited onto the pipe wall, was found to increase across experiments F2C1 and F3C1 where the fluid velocity was increased to 0.2 m s^-1^ and 0.3 m s^-1^ (Fig. 7a). The decay coefficient was found to be constant at 0.006 s^-1^ for the experiments F1C1, F1C2, and F1C3 where fluid velocity was held constant at 0.07 m s^-1^ (Fig. 7b). This suggests that a faster deposition occurred in the experiments with higher fluid velocities, but a smaller DP fraction led to lower material loads. Nevertheless, Fig. 6b shows that 80% of all settable material was deposited in the first 15 m of the pipe in the F1C1 experiment, where the same fraction was deposited in the first 25 m of pipe in the F2C1 and F3C1 experiments.

### 3.4 Microscopy analysis

**Fig. 8:**
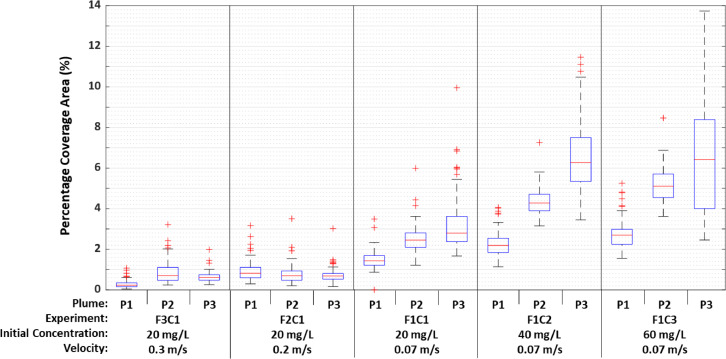
Percentage coverage area of brightfield microscopy images of pipe coupon wall samples collected from the invert position of the coupon section, after the passage of plumes P1, P2 and P3 in each experiment. Boxplots were based on data of 100 field of views observed in each sample surface using automated brightfield microscopy.

Brightfield microscopy was also used to characterize the occurrence of particle attachment on the pipe wall at a pipe section located relatively far from the injection point. Even though only a negligible fraction of DP particles was able to reach the coupon section located at pipe length of 106 m from the pipe loop inlet (Figs. 1 and 6), a consistent number of iron oxide particle deposits were observed at the pipe invert. Pipe coupon samples from the obvert location were not analyzed in this study since previous research provided strong evidence that only negligeable quantities of particles accumulate at the pipe obvert (Braga and Filion, 2021). Fig. 8 indicates box-and-whisker plots for the percentage coverage area of iron oxide particles detected on the coupon samples across a total of 100 field of views (FOVs) per coupon sample for each individual sediment plume. The results in Fig. 8 indicate that the percentage coverage area was increased with the passage of each sediment plume in the low-velocity experiments F1C1, F1C2, and F1C3, and that this increase in percent coverage was more noticeable with an increase in the injection concentration. In contrast, the percent coverage area for samples collected after the third plume in the high-velocity experiments (F2C1 and F3C1) was smaller than the percent coverage area observed in the low-velocity experiments (F1C1, F1C2 and F1C3, Fig. 8). Furthermore, no changes to the percent coverage area were detected between the consecutive sediment plumes (P1, P2 and P3) for the high-velocity experiments (F2C1 and F3C1), while consistent increases in percent coverage area were detected in each plume for the low-velocity experiments (F1C1, F1C2 and F1C3). Experiments F1C1, F1C2, and F1C3 performed with a fluid velocity of 0.07 m s^-1^ resulted in a deposition of larger particle sizes on the pipe coupons. Previous data from SSC samples and the PSD analysis showed that particle sizes in the range of 4.6 µm to 26 µm are likely candidates to form deposits rather than their smaller-sized counterparts that remain in suspension. In comparison, the smaller particle sizes found on the pipe coupon samples may indicate that a different mechanism of particle deposition occurred in the pipe. The new deposition mechanism, however, only targets a very small fraction of the suspended particles whose sizes are compatible with the “valleys” and occlusions that make up the absolute roughness of the PVC pipe walls.

**Fig. 9:**
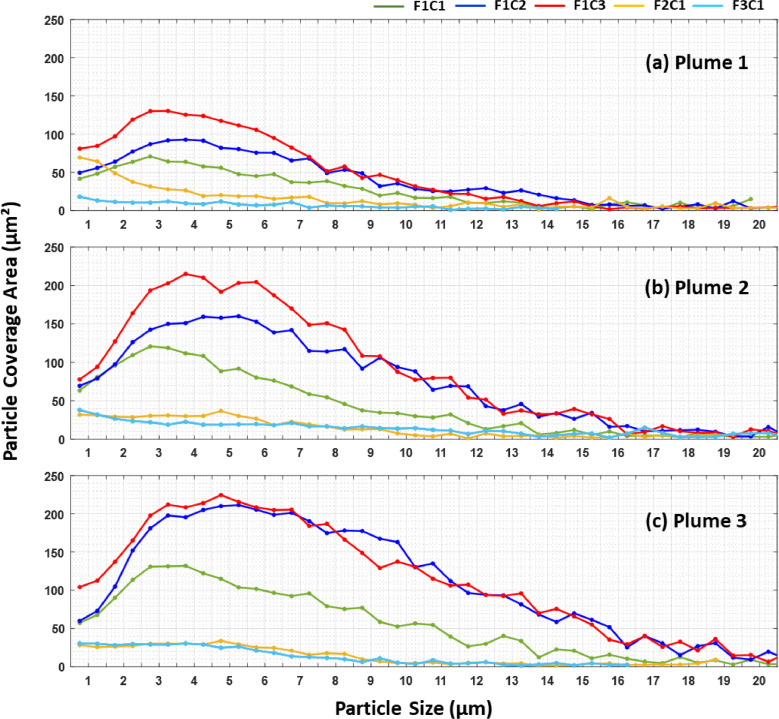
Particle coverage area per particle size bin of brightfield microscopy images taken from pipe wall samples collected after the passage of the (a) first plume, (b) second plume, and (c) third plume in each experiment. All samples were collected at the invert position of the pipe loop coupon section.

To confirm the hypothesis regarding the particle sizes of deposits found on the pipe wall samples, an analysis of the particle size distribution of the microscopic images was also performed. Fig. 9 plots particle coverage area as a function of particle size detected with brightfield microscopy after the passage of each sediment plume. The data in Fig. 9c shows that as the injection concentration was increased from 20 mg L^-1^ (F1C1) to 60 mg L^-1^ (F1C3), the dominant particle size increased from 3 μm to 5 μm. Fig. 9 also shows clearly that as the injection concentration was increased across experiments, a substantial increase in particle coverage area was observed for all particle sizes ranging from 1 μm to 20 μm after the passage of the second and third plumes. The particle coverage area for the F2C1 and F3C1 experiments remained below 50 μm² per particle size bin, with a dominant particle size smaller than 5 μm.

## 4 Discussion

The experimental results presented in this paper demonstrate that it is possible to quantify the total load of material deposits along a pipe section using turbidity data calibrated with SSC samples. However, the relationship between turbidity and SSC can change rapidly based on the PSD of suspended sediments. This means that regression coefficients should be determined for specific systems and particle properties.

Large errors in the prediction of material deposition can be expected without an adequate assessment of regression coefficients to transform turbidity data into SSC. In the experiments of this paper, regression coefficients with a small numerical value seem to have been produced by the lower fluid velocities in comparison to the flush velocities previously used to assess these.

The experiments performed showed that suspended iron oxide particles as small as 4.6 μm can be rapidly deposited on the pipe wall under common operational conditions in drinking water systems. The formation of material deposits was mostly affected by the SSC at the injection point, and more specifically, by the DP fraction of the initial SSC that was comprised of 69% to 75% of the total mass of particles injected into the pipe loop. Data also showed that 90% of the DP fraction were attached to the pipe wall along the first 40 m of pipe length in the loop, while the deposition rate rapidly decreased along the length of the pipe according to a first-order decay process. Increasing the fluid velocity from 0.07 m s^-1^ to 0.3 m s^-1^ (0.3 m s^-1^ is often considered a self-cleaning velocity, Blokker et al. (2011)), produced a decrease of the TDL of 6%, and an increase of the *particle size threshold* that defines the DP and NDP fractions of the initial SSC. While it is clear that increasing fluid velocity has an impact on the *particle size threshold*, it is still unclear how small particle sizes in suspension are rapidly deposited onto the pipe walls under turbulent flow conditions (Boxall and Saul, 2005). Previous research suggested multiple mechanisms that might explain how particles reach the pipe walls while in suspension in turbulent flow (van Thienen et al., 2011), but their attachment mechanisms and origins of adhesion forces are still poorly understood.

The fact that material deposits on the pipe wall were formed by the larger particle fractions of the initial SSC and that the PST increased with fluid velocity suggests that the *gravitational settling* mechanism might be driving the particle deposition process. This hypothesis is also supported by the fact that no noticeable quantity of particles was observed to accumulate and adhere to the pipe obvert in previous experiments (Braga and Filion, 2021). In this context, it is possible that current methods used to forecast the settling of suspended particles might not be appropriate for drinking water pipes due to fundamental differences of particle attachment mechanisms that are still poorly understood. Therefore, further research is required to: (1) confirm the processes that govern the possible gravitational settling of small particles in drinking water systems, and (2) gain a better understanding of the effect of particle adhesion to the pipe wall in these deposition processes.

In addition, the smaller differences between F2C1 and F3C1 in comparison to F1C1 and F2C1 seem to indicate a tapering of particle attachment effects at higher velocities. This seems to be caused by the decreasing pool of available suspended particles at larger particle sizes (Fig. 5). Peaks of particle size distribution in Fig. 5 shows that most of the particle mass is concentrated around 2 µm – 3 µm, while the analysis of the experimental data suggests that a particle size threshold (assumed as the d80 in the curves) increased from 4.6 µm to 6.8 µm with the fluid velocity. Therefore, the mass of particles larger than the threshold rapidly decreased with the increase in the particle size threshold. Further increasing the flow rate would continuously increase the particle size threshold and reduce the DP fraction until the threshold reached the maximum particle size and particle deposition would stop altogether. However, the microscopy data collected at the pipe wall samples located at pipe length of 106 m – which for the current experiments had a negligible mass in comparison to the injected SSC – seems to suggest that a different wall-attachment process operates on the particles. In this case, the microscopy data analysis suggested that increasing the fluid velocity decreased the accumulation rate of larger particles, while only smaller particles were able to remain attached to the pipe wall. The similarity in the percent coverage area between F2C1 and F3C1 suggests that particles may have occupied all remaining roughness ‘valleys’ on the pipe wall available for the attachment of selected fine particles of compatible size and inhibited any additional attachment of particles to the pipe wall. This might be indicative that the self-cleaning velocity concept is effective in inhibiting this second attachment mechanism, which has the potential to dominate the particle attachment process in operational DWDSs where larger particles remain immobile due to low fluid velocities during normal flow conditions. In addition to this, the similarity in the percent coverage area between the three sediment plumes of the F2C1 and F3C1 experiments further suggests that all roughness ‘valleys’ may have been occupied by particles after the first plume which may have inhibited any additional particle accumulation in the second and third plumes of these experiments.

### 4.1 Experimental limitations

The experiments performed mimicked the acute loading of suspended particles from an upstream turbidity event (Sunny et al., 2020), in a downstream pipe under more normal daily conditions. The experiments used simplified steady state conditions, while daily demand patterns are known to have an impact on material accumulation (Husband et al., 2008), these experiments were arguably performed under the highest daily peak flow which was greater than the self-cleaning velocities (Blokker et al., 2011). Despite this, particle accumulation was strongly evident. It is also important to highlight that the acute and rapid process of particle attachment to the pipe wall only represents a fraction of the material accumulation processes that occur inside DWDSs. The experiments made use of relative new PVC pipes free of biofilms. In operational systems, pipe materials have undergone changes and are populated with biofilm layers which likely play a role in the attachment dynamics of suspended particles. A single pipe diameter was tested in the experiments whereas operational DWDSs have a large range of pipe diameters. It is expected that the pipe diameter effects mostly translate to impacts on fluid velocity and turbulence structures. Another key simplification consisted of the use of suspended particles with a stable density, which greatly facilitated the analysis of particle size distributions. Both particle size and density are key to determine particle transport in suspension by turbulent flows (Guha, 2008). In the case of operational DWDSs, such analysis is more challenging since a large range of particle types with multiple densities are expected from pipe wall material deposits. Additional biochemical processes that are responsible for the transformations between dissolved and particulate matter were also avoided in the experiments, but they are commonly reported in DWDSs, including the precipitation of inorganic matter and growth of biofilms.

Nonetheless, only inorganic suspended sediments were considered here, while previous research has shown that organic matter comprises a significant fraction of discolouration materials (Carrière et al., 2005; Husband and Boxall, 2011; Mussared et al., 2019). The combination of organic and inorganic matter adds further complexity to the problem, since organic matter particles can rapidly change their size and density due to smaller cohesion forces and heterogeneous composition. Organic matter can also attach to inorganic particles and can change their density, have an impact on the drag force of the flow, and improve their capability to adhere to a solid surface.

These important simplifications adopted here limits the direct applicability of the research findings, but also highlights the complexity of the material accumulation phenomena in DWDSs. Further research is required to properly address each one of these aspects, while experiments under controlled conditions are recommended in order to differentiate the multiple processes under investigation.

## 5 Conclusion

A series of experiments were performed to evaluate the interplay between the SSC and the fluid velocity in the process of particle deposition during the passage of a highly concentrated plume of particles. It was found that the magnitude of the SSC influenced the formation of deposits and was directly proportional to the total deposited load in the pipe loop. By contrast, the fluid velocity was inversely proportional to the total deposited load, but increasing the velocity from 0.07 m s^-1^ to 0.3 m s^-1^ produced a 6% reduction in the fraction of particles deposited on the pipe walls. A similar change of 6% was also observed by analyzing the particle size data for the fraction of injected particles that reached the end of the pipe loop. This suggests that the increase in velocity inhibited the deposition of particles in the size range of 4.6 μm to 6.8 μm.

In addition, the fitting of a first-order exponential decay model to the SSC data also showed that a particle size threshold (PST) divided the initial injected particles into two fractions: (i) a fraction of deposited particles (DP), and (ii) a fraction of non-deposited particles (NDP), and that only the concentration corresponded to the DP fraction decay along the plume passage. The exponential decay coefficients increased with the fluid velocity, but the fraction of DP decreased with an increase in fluid velocity. Due the fast deposition of the DP, most of the particles were deposited in the initial sections of the pipe loop. In contrast, the observations of pipe wall samples with brightfield microscopy collected far away from the particle injection position revealed that small particles with a dominant particle size of 5 µm were able to attach to the pipe wall.

The experimental data suggests that the gravitational settling of particles might explain the fast deposition of DP fraction, while the observations of particles on the pipe coupon samples might indicate that another particle attachment mechanism with substantially smaller deposition rate might also occur. The presence of particle deposits from previous plumes had a negligible impact on the particle deposition rate and attachment in the initial pipe sections. But considering the second mechanism, the microscopy data suggested that at high fluid velocities there was little difference in particle deposition between plumes, whereas at lower fluid velocities, the particle deposition increased with each successive plume. Further studies are required to clarify the complete mechanics of the observed phenomena, as well as to address several shortcomings and simplifications of the experiments proposed in this research.

## Declaration of Competing Interest

The authors declare no conflict of interest.
